# Phenotypic heterogeneity of giant cell arteritis in an Asian cohort: clinical, imaging, and laboratory characteristics

**DOI:** 10.3389/fmed.2026.1812045

**Published:** 2026-06-30

**Authors:** Xin Dang, Qingqing Zhu, Ruihuan Shen, Yongmei Han

**Affiliations:** 1 Department of Rheumatology, Sir Run Run Shaw Hospital, School of Medicine, Zhejiang University, Hangzhou, China; 2 Department of Radiology, Sir Run Run Shaw Hospital, School of Medicine, Zhejiang University, Hangzhou, China; 3 Department of Translational Medicine and Clinical Research, Sir Run Run Shaw Hospital, Zhejiang University School of Medicine, Hangzhou, China

**Keywords:** Asian people, biomarkers, classification, diagnostic imaging, giant cell arteritis, phenotype

## Abstract

**Background:**

Giant cell arteritis (GCA) exhibits phenotypic heterogeneity. The diagnosis of large vessel involvement in GCA remains challenging, and conventional inflammatory markers have limitations. This study aimed to characterize phenotypic profiles and explore the role of novel hematological indices in an Asian cohort.

**Methods:**

This single-center retrospective study enrolled 110 patients diagnosed with GCA between 2015 and 2025. Patients were classified into cranial (cGCA), mixed (mixGCA), and isolated large-vessel (LV-GCA) phenotypes. Demographics, clinical features, and laboratory parameters—including the systemic immune-inflammation index (SII), neutrophil-to-lymphocyte ratio (NLR), platelet-to-lymphocyte ratio (PLR), and serum albumin—were analyzed. The performance of the 1990 ACR and 2022 ACR/EULAR classification criteria was evaluated.

**Results:**

The cohort comprised 110 patients (65 men, 59.1%; 45 women, 40.9%), and was divided into cGCA (44.5%), mixGCA (36.4%), and LV-GCA (19.1%). LV-GCA presented with more constitutional symptoms and limb claudication but fewer cranial symptoms. Novel hematological indices, particularly SII and PLR, showed significant positive correlations with C-reactive protein levels. Multivariate logistic regression analysis revealed that serum albumin level was independently associated with large-vessel involvement (LVI; OR = 0.865, 95% CI [0.767–0.974], *p* = 0.017). The 2022 ACR/EULAR criteria had higher overall sensitivity (77.3%) than the 1990 ACR criteria (69.1%).

**Conclusion:**

GCA presents a distinct clinical profile in this Asian cohort. Novel hematological indices (including SII and PLR) correlate with acute-phase reactants, and serum albumin is associated with LVI. These preliminary findings suggest that these biomarkers, alongside lower-limb arterial ultrasound, may aid in identifying LVI, requiring prospective validation.

## 1 Introduction

Giant Cell Arteritis (GCA) is a systemic vasculitis predominantly affecting medium and large vessels in adults aged > 50 years. Epidemiologically, the disease exhibits striking geographical variation, with the highest incidence in Northern European populations (up to 20–30 cases per 100,000 person-years), while historically lower rates reporting in Asian cohorts ( < 1 per 100,000) (1).

Classic or cranial GCA (cGCA) typically presents with headache, sudden visual loss, and temporal artery abnormalities. Although the 1990 American College of Rheumatology (ACR) classification criteria demonstrate good sensitivity for cGCA (2, 3), they fail to adequately identify patients with predominant large-vessel GCA (LV-GCA) (4). Advances in imaging modalities, including ultrasound and 18F-fluorodeoxyglucose positron emission tomography-computed tomography (18F-FDG PET-CT), have significantly refined diagnostic accuracy and are formally incorporated into the 2022 ACR/European League Against Rheumatism (EULAR) classification criteria, though further validation in diverse real-world populations remains necessary (5).

In contrast, LV-GCA often manifests with a more systemic phenotype and higher relapse rates, posing a distinct diagnostic challenge (6). Although temporal/axillary artery ultrasound and PET-CT are now integrated into diagnostic pathways, the potential utility of lower limb arterial ultrasound, as an adjunctive tool, warrants further exploration. Beyond imaging, conventional inflammatory markers have inherent limitations in predicting disease activity, flares and phenotypic distinctions. Notably, in other systemic inflammatory diseases, such as systemic Lupus Erythematosus (SLE), rheumatoid arthritis (RA), and adult-onset still’s disease (AOSD), novel hematological indices derived from routine blood tests have shown promise as biomarkers (7). Correspondingly, there is a critical need to identify more reliable biomarkers, including indices like the Systemic Immune-Inflammation Index (SII, which was calculated as platelet count × neutrophil count/lymphocyte count, using peripheral blood cell counts obtained from routine complete blood count; all expressed as × 10^9^/L) to facilitate risk stratification and predict specific disease features like large-vessel involvement (LVI) in GCA.

The pathogenesis of GCA involves a breakdown in immune tolerance, leading to vascular inflammation driven by tissue-resident CD4 + T cells and macrophages (8).

The preliminary evidence indicated that allogeneic T cells exhibit differential response patterns when interacting with various arteries in same GCA donor (9). Therefore, we hypothesize that the distinct clinical phenotypes of GCA may be driven by artery-specific immunological microenvironments that shape localized T-cell recruitment, activation, and effector responses.

Building on these unmet needs, this study aimed to address three key objectives: 1) To systematically characterize the clinical, laboratory, and imaging profiles of cGCA, LV-GCA, and mixed GCA (mixGCA) subgroups in a real-world cohort, with a focus on differentiating features that aid early diagnostic stratification; 2) To evaluate the performance of the 1990 ACR and 2022 ACR/EULAR classification criteria across these phenotypic subgroups, particularly their sensitivity for detecting LV-GCA; 3) To explore the utility of novel hematological biomarkers (including the SII, neutrophil-to-lymphocyte ratio [NLR], platelet-to-lymphocyte ratio [PLR], and serum albumin) in identifying LVI. We hypothesized that: (1) cGCA, LV-GCA, and mixGCA would exhibit distinct clinical and imaging signatures; (2) the 2022 ACR/EULAR criteria would demonstrate improved overall sensitivity compared to the 1990 ACR criteria but remain suboptimal for LV-GCA; and (3) novel hematological indices, especially SII and serum albumin, would show significant associations with LVI and thus could serve as practical biomarkers for risk stratification in routine clinical practice.

## 2 Materials and methods

### 2.1 Study design and eligibility criteria

The study was performed under routine clinical practice conditions. We retrospectively reviewed databases to identify patients with a clinical diagnosis of GCA at Sir Run Run Shaw Hospital between January 2015 and June 2025.

The golden standard for the diagnosis of GCA was the judgment of two experienced clinicians after a follow-up of at least 6 months, considering all available information (clinical data, biochemical, radiological, and temperoal artery biopsy (TAB) results, and evolution during follow-up).

The main features were as follows: (1) age at disease onset ≥ 50 years; (2) the presence of associated clinical symptoms or signs: cranial ischemic symptoms (headache, scalp tenderness, abnormal temporal artery examination, jaw claudication, visual loss), polymyalgia rheumatica (PMR), constitutional symptoms, and manifestations related to extracranial LVI (limb claudication, pulse discrepancy, bruits of extra-cranial arteries unrelated to atherosclerosis, tenderness to palpation or decreased pulsation); (3) raised acute-phase reactants (erythrocyte sedimentation rate [ESR] > 50 mm/h or C reactive protein [CRP] > 6 mg/L); (4) at least one positive results in an objective diagnostic test: vasculitis based on the TAB (characterised by a predominance of mononuclear cell infiltration or granulomatous inflammation, with or without the presence of multinucleated giant cells), the presence of a “halo sign” (the halo sign was defined as: homogeneous, hypoechoic wall thickening, well delineated toward the luminal side, visible in both longitudinal and transverse planes, most commonly concentric in transverse scans, a wall thickness ≥ 0.5 mm was considered positive for the halo sign) and/or “compression sign” (the compression sign was defined as: gentle pressure was applied using the ultrasound transducer until the lumen of the temporal artery was occluded (no pulsation visible on B-mode). The compression sign was considered positive if the arterial wall remained visible in transverse view, presenting as an echogenicity distinct from the surrounding tissue due to mural inflammation) on a color Doppler ultrasound (CDUS) of the temporal and/or axillary arteries in the absence of atherosclerosis, or evidence of large vessel vasculitis on computed tomography angiography (CTA), or on 18F-FDG PET-CT; (5) prompt and persistent response to glucocorticoid therapy; and 6) no change of diagnosis during a follow-up of at least 6 months.

Patients were excluded if they had: active malignancies (within the preceding 5 years); current systemic infections (viral, bacterial or fungal); other systemic autoimmune diseases (e.g., RA, Polyarteritis Nodosa, etc.); recent major surgery/trauma ( < 3 months); or incomplete medical records.

### 2.2 Phenotypic subgroup classification

Patients were classified into three subgroups based on clinical and imaging features:

(i) cGCA: Defined by the presence of cranial ischemic symptoms and/or abnormalities of the temporal arteries on physical examination; and evidence of vasculitis on the TAB or temporal artery imaging (US, PET-CT); No evidence of LVI on adequately performed large vessel imaging (18F-FDG PET/CT, CTA, MRA or US). Note: If large-vessel imaging is not performed, classification as cGCA is considered provisional.

(ii) LV-GCA: Defined by imaging evidence of vasculitis affecting the aorta and/or its major branches on any of the following: 18F-FDG PET-CT scan (Vascular FDG uptake higher than liver); CTA (diffuse, concentric mural thickening), and large vessel US: anonymous artery, common carotid artery, subclavian artery, axillary artery, humeral arteries, brachial artery, radial artery, ulnar artery, iliac artery, femoral artery, popliteal artery (A hypoechoic, circumferential “halo” sign), not attributable to atherosclerosis, no evidence of cranial arteritis.

(iii) MixGCA: Defined by the concurrent presence of cranial artery involvement (defined as: cranial ischemic symptoms and/or abnormalities of the temporal arteries on physical examination; and evidence of vasculitis on temporal artery biopsy or temporal artery imaging); and LVI (defined as: imaging evidence of vasculitis affecting the aorta and/or its major branches on PET-CT, CTA, MRA, or large vessel US);

### 2.3 Imaging protocols

Ultrasound examinations of the arteries were conducted by specialized sonographers with at least 5 years’ experience in the evaluation of vasculitis, with 18 MHz hockey stick transducers. Only those meeting diagnostic quality criteria were included (presence of a “halo sign” and/or “compression sign” on a color Doppler ultrasound (CDUS) of the temporal arteries, not attributable to atherosclerosis). We applied standard linear transducers (frequency range 12–15 MHz) on large vessels (eg. anonymous artery, common carotid artery, subclavian artery, axillary artery, humeral arteries, brachial artery, radial artery, ulnar artery, iliac artery, femoral artery, popliteal artery).

### 2.4 Data collection

Demographic, clinical, and laboratory data were extracted from medical records. Collected variables included:

Demographics: Age at onset, sex, interval from symptom onset-to-diagnosis, and initial referring specialty.

Clinical Features at Onset: New-onset headache, jaw claudication, scalp tenderness, transient or permonant visual loss (amaurosis fugax), stroke, transient ischemic attack (TIA), arthralgia/myalgia, PMR, fatigue, fever, anorexia, weight loss, night sweats, limb claudication.

Laboratory Parameters at Diagnosis: Erythrocyte sedimentation rate (ESR), C-reactive protein (CRP), haemoglobin (Hb), creatinine, albumin, blood lipids profiles, alkaline phosphatase (ALP), red cell distribution width (RDW), NLR, PLR, and SII.

Treatment: Initial and follow-up treatment strategies, including glucocorticoid (GC) dosing regimens and use of adjunctive immunosuppressive agents.

Severe Ischemic Manifestations: Defined as the presence of at least one of the following features at diagnosis: jaw claudication, permanent visual loss, or cerebrovascular accident.

### 2.5 Definition of relapse

Disease relapse was defined as the recurrence or worsening of GCA-attributable symptoms accompanied by an elevation in acute-phase reactants (ESR/CRP), occurring during glucocorticoid taper or within 3 months of glucocorticoid discontinuation.

### 2.6 Statistical analysis

All statistical analyses were performed using SPSS software (version 25.0). A two-sided *p* < 0.05 was considered statistically significant. Continuous variables are presented as mean ± standard deviation (SD) or median with interquartile range (IQR), and were compared using the Kruskal-Wallis test. Categorical variables are presented as counts and percentages and were compared using Fisher’s exact test.

Binary logistic regression was used to identify factors independently associated with GCA disease subtypes. Correlations between disease activity markers and hematological indices (NLR, PLR) were assessed using Spearman’s rank correlation coefficient.

## 3 Results

### 3.1 Baseline demographic and clinical characteristics for GCA patients

As shown in Table 1, the cohort consisted of 110 patients with GCA (65 men and 45 women), with a male-to-female ratio of 1.44. Infectious diseases specialists were the most frequent initial consulting physicians. The median age at disease onset was 71 years (IQR: 66–76). Stratified by age at diagnosis, 19 patients (17.3%) were ≤ 64 years, 77 patients (70.0%) were 65–79 years, and 14 patients (12.7%) were ≥ 80 years.

**TABLE 1:** Basic characteristics of giant cell arteritis patients and differences by sex.

Characteristic	Total (*n* = 110)	Female (*n* = 45)	Male (*n* = 65)	*P-*value
Age, mean ± SD	71 ± 7	73 ± 8	69 ± 8	**0.031**
Typing (cGCA/LV-GCA/mixGCA)	49/21/40	24/4/17	25/17/23	0.060
Current smoker	20 (18.2)	1 (2.2)	19 (29.2)	**<0.001**
Initial Provider, n (%)				**0.011**
Infection specialist	63	18 (40.0)	45 (69.2)	
Rheumatologist	30	19 (42.2)	11 (16.9)	
Others	17	8 (17.8)	9 (13.8)	
BMI (kg/m^2^) (mean ± SD)	22.13 ± 3.35	21.69 ± 3.43	22.43 ± 3.28	0.266
Time from symptom onset to diagnosis, n (%)				**0.013**
<1 month	66 (60)	21	45	
1–3 months	32 (29.1)	15	17	
≥3 months	12 (10.9)	9	3	
Symptoms at diagnosis, n (%)				
Coexisting PMR symptoms	32 (29.1)	15 (33.3)	12 (18.5)	0.114
Cranial symptoms	75 (68.2)	34 (75.6)	41 (63.1)	0.167
Morning stiffness	15 (13.6)	11 (24.4)	4 (6.2)	**0.009**
Constitutional symptoms	93 (84.5)	34 (75.6)	59 (90.8)	**0.035**
Fever	93 (84.5)	34 (75.6)	60 (92.3)	0.065
Night sweats	8 (7.3)	3 (6.7)	5 (7.7)	0.839
Fatigue	32 (29.1)	14 (31.1)	18 (27.7)	0.831
Anorexia	26 (23.6)	9 (20.0)	17 (26.2)	0.501
Weight loss	27 (24.5)	12 (26.7)	15 (23.1)	0.656
Limb claudication	13 (11.8)	4 (6.2)	9 (13.8)	0.623
Laboratory tests, mean ± SD				
CRP, mg/L	111.5 ± 62.2	87.1 ± 58.3	128.4 ± 59.6	**<0.001**
ESR, mmHg/1st h	94 ± 31	94 ± 32	94 ± 30	0.899
RDW (%)	13.3 ± 1.23	13.56 ± 1.05	13.8 ± 1.31	**0.043**
Platelet count ( × 10^9^/L)	371 ± 131	381 ± 128	365 ± 134	0.537
NLR	6.67 ± 5.45	6.15 ± 2.01	7.26 ± 5.81	0.165
PLR	322.45 ± 175.02	315.62 ± 152.66	327.07 ± 189.68	0.739
SII	2572.60 ± 2475.11	2278.49 ± 1951.76	2771.69 ± 2771.07	0.310
Albumin (g/L)	31.6 ± 4.2	33.5 ± 3.4	30.3 ± 4.2	**<0.001**
Creatinine (μmol/L)	65.0 ± 15.7	56.7 ± 9.6	70.6 ± 16.6	**<0.001**
ALP (U/L)	145 ± 100	109 ± 39	170 ± 120	**0.002**
Total cholesterol (mmol/L)	3.93 ± 2.57	4.35 ± 1.02	3.58 ± 0.84	**<0.001**
Positive US of cranial arteries*^a^*, *n* (%)	40/104 (38.5)	19/43 (44.2)	21/61 (34.4)	0.542
Positive US of axillary arteries*^a^*, *n* (%)	11/92 (12)	5/41 (12.2)	6/51 (11.8)	0.212
Positive US of lateral femoral circumflex artery*^a^*, *n* (%)	23/70 (32.9)	7/31 (22.6)	16/33 (48.5)	0.245
Positive PET/CT findings, *n* (%)	53/61 (86.9)	19/20 (95.0)	37/41 (90.2)	0.153
Histologically confirmed arteritis	14/27 (51.9)	7/10 (70.0)	7/17 (41.2)	0.277
Management				
GCs	102 (92.7)	41	61	0.590
MTX	70 (63.6)	26	42	0.778
Cerebral infarction or TIA	2 (1.8)	1 (2.0)	1 (1.5)	1.000

mean **±** SD, mean ± standard deviation; IQR, interquartile range; Statistical tests: Continuous variables analyzed by Kruskal-Wallis H test; categorical variables analyzed by Fisher’s exact test; PMR symptoms, polymyalgia rheumatica symptoms (includes neck and shoulder pain, and/or pelvic girdle pain). cGCA, cranial giant cell arteritis; LV-GCA, large-vessel giant cell arteritis; mixGCA, mixed giant cell arteritis; CRP, C-reactive protein; ESR, erythrocyte sedimentation rate; RDW, red cell distribution width; NLR, neutrophil-to-lymphocyte ratio; PLR, platelet-to-lymphocyte ratio; SII, systemic immune-inflammation index (SII was calculated as platelet count × neutrophil count / lymphocyte count, using peripheral blood cell counts obtained from routine complete blood count; all expressed as × 109/L); ALP, alkaline phosphatase; BMI, Body Mass Index; LDL, low-density lipoprotein; US, ultrasound; PET/CT, positron emission tomography/computed tomography; GC, Glucocorticoids; MTX, Methotrexate; TIA, Transient ischemic attack.

*^a^*Defined as the presence of a halo sign and/or compression sign in at least one cranial artery/axillary arteries/lateral femoral circumflex artery. Values in bold indicate statistical significance (*P* < 0.05).

Constitutional symptoms were the most common presenting feature (89.1%). Cranial manifestations included new-onset headache (68.2%), scalp tenderness (37.3%), and jaw claudication (19.1%). Cerebrovascular ischemic events occurred in only 2 patients. Limb claudication was reported by 13 patients (11.8%) during the disease course. Twenty (18.2%) patients were current smokers. The mean ESR was 94 mm/h, and the mean CRP was 111.5 mg/L. Additional baseline laboratory parameters are summarized in Table 1.

Vascular ultrasound was performed across multiple territories, including the temporal, common carotid, and subclavian arteries, as well as the anonymous artery, and bilateral upper/lower limb arteries. The detection rates varied among different blood vessels: temporal arteries (38.5%), lower limb arteries (32.9%), and axillary arteries (12.0%). The temporal artery halo sign was present unilaterally in 10 patients and bilaterally in 30 patients, accounting for 36.4% in the cohort. Of 61 patients who underwent 18F-FDG PET-CT, 53/61 (86.9%) showed increased metabolic activity in the aorta or its primary branches. The most frequently involved vessels were the iliac (28.2%) and femoral (27.3%) arteries, followed by the aortic arch/brachiocephalic trunk, carotid arteries, and abdominal aorta (20.9% each). Ultrasound-guided temporal artery biopsy was performed in 27 patients, with positive results in 14 (51.9%) patients. Supplementary Figure 1 displayed the detailed sample size selection in ultrasound.

Regarding treatment, 102 patients (92.7%) received prednisone. Methotrexate was the most commonly used glucocorticoid-sparing agent initiated early in the disease course (63.6%). The relapse rate was approximately 12%. Biologic agents were added for disease relapse in only two patients.

### 3.2 Clinical manifestations and serum parameters of GCA patients with different phenotypes

Based on integrated clinical and imaging assessments, patients were categorized into three phenotypic subgroups: cGCA (44.5%, *n* = 49), mixGCA (36.4%, *n* = 40), and isolated LV-GCA (19.1%, *n* = 21). The distribution of demographic, clinical, and diagnostic features across these subtypes was summarized in Table 2.

**TABLE 2:** Univariate comparison of characteristics among different subsets of giant cell arteritis.

Characteristics	cGCA (*n* = 49)	LV-GCA (*n* = 21)	mixGCA (*n* = 40)	*P-*value
Age, mean ± SD	72 ± 7	67 ± 8	72 ± 9	0.071
Male, *n* (%)	25 (51.0)	17 (81.0)	23 (57.5)	0.063
Current smoker	8 (16.3)	4 (19.0)	8 (20.0)	0.899
BMI (kg/m^2^) (mean ± SD)	21.3 ± 3.1	22.8 ± 3.4	22.7 ± 3.5	0.096
Initial Provider, *n* (%)				0.091
Infection specialist	23 (46.9)	15 (71.4)	25 (62.5)	
Rheumatologist	14 (28.6)	6 (28.6)	10 (25)	
Other	12 (24.5)	0 (0)	5 (12.5)	
Symptoms at diagnosis, n (%)				
New-onset headache	43 (87.8)	2 (9.5)	30 (75)	**< 0.001**
Scalp tenderness	24 (49.0)	0 (0)	17 (42.5)	**< 0.001**
Visual loss	1 (2.0)	0 (0)	1 (2.5)	1.000
Jaw claudication	9 (18.4)	0 (0)	12 (30)	**0.018**
PMR symptom	16 (32.7)	5 (23.8)	11 (27.5)	0.728
Constitutional symptoms	35 (71.4)	21 (100.0)	39 (97.5)	**0.002**
Fever	40 (81.6)	19 (90.5)	39 (97.5)	**0.026**
Limb claudication	2 (4.1)	5 (23.8)	6 (15)	**0.037**
Laboratory tests, mean ± SD				
CRP (mg/L)	102 ± 68	113 ± 53	122 ± 58	0.352
ESR (mmHg/1st h)	89 ± 33	92 ± 32	101 ± 26	0.195
RDW (%)	13.4 ± 1.2	12.9 ± 1.0	13.3 ± 1.4	0.319
Platelet count ( × 10^9^/L)	353 ± 123	348 ± 121	406 ± 142	0.115
NLR	6.96 ± 7.31	6.18 ± 2.98	6.56 ± 3.45	0.851
PLR	319.24 ± 205.20	301.57 ± 182.64	337.73 ± 125.78	0.740
SII	2709.19 ± 3362.70	2229.87 ± 1450.86	2585.54 ± 1389.52	0.762
Albumin (g/L)	33.1 ± 3.9	30.5 ± 4.5	30.3 ± 3.9	**0.006**
Creatinine (μmol/L)	64 ± 16	75 ± 18	62 ± 11	**0.011**
Prednisone	43 (87.8)	20 (95.2)	39 (97.5)	0.211
MTX	23 (46.9)	16 (76.2)	31 (77.5)	**0.005**
Tocilizumab	1 (2.0)	0 (0)	1 (2.5)	1.000

cGCA, cranial giant cell arteritis; LV-GCA, large-vessel giant cell arteritis; mixGCA, mixed giant cell arteritis. Other abbreviations are the same as in Table 1. Statistical tests: Continuous variables were analyzed using Kruskal-Wallis H test as appropriate; categorical variables were analyzed using Fisher‘s exact test. Values in bold indicate statistical significance (*P* < 0.05).

The proportion of male patients was higher in the LV-GCA subgroup (81%) than in the cGCA subgroup (51%) and mixGCA subgroup (57.5%), although the difference was not statistically significant (*p* = 0.063). Age at onset did not differ significantly among groups (*p* = 0.071), although patients with LV-GCA were numerically younger than those with cGCA and mixGCA. Regarding referring specialty, infectious disease specialists diagnosed the majority of LV-GCA (71.4%) and mixGCA (62.5%) cases, while rheumatologists diagnosed 25.0–28.6% of cases in each subgroup.

The main manifestations in the three subtypes were compared in different aspects. The frequency of concomitant PMR was comparable among the three phenotypes. Cranial symptoms, constitutional symptoms, and lower limb claudication showed a prominent differential distribution among the three groups.

Cranial symptoms were rare or absent in the LV-GCA group (9.5%, *p* < 0.001 for headache; 0%, *p* = 0.018 for scalp tenderness; and 0%, *p* = 0.018 for jaw claudication). Constitutional symptoms were present in all LV-GCA (100%) and most mixGCA (97.5%) patients, compared to 71.4% in cGCA patients (*p* = 0.002). Limb claudication was significantly more frequent in LV-GCA and mixGCA than in cGCA (*p* = 0.037).

Laboratory tests were compared between the three groups, only serum albumin and creatinine levels were significantly different (*p* = 0.006 and *p* = 0.011). The significant differences observed in univariate analysis did not persist after adjustment in multivariate logistic regression analysis. Accordingly, we merged the LV-GCA and mixed GCA subgroups, and further compared GCA patients with and without LVI in Table 3. In univariate analysis, cranial symptoms, limb claudication, constitutional symptoms, and BMI showed significant differences. After multivariate adjustment, only serum albumin (OR = 0.865, 95% CI [0.767–0.974], *p* = 0.017) and cranial symptoms (OR = 0.303, 95% CI [0.101–0.909], *p* = 0.033) were independently associated with LVI.

**TABLE 3:** Univariate and multivariate logistic regression analysis of factors associated with large vessel involvement in giant cell arteritis patients.

Characteristics	GCA without LVI (*n* = 49)	GCA with LVI (*n* = 61)	Univariate *P-*value	Multivariate OR (95% CI)	Multivariate *P-*value
Age, mean ± SD	72 ± 7	70 ± 9	0.206	–*	–
Male, n (%)	25 (51.0)	40 (65.6)	0.172	–	–
Current smoker	8 (16.3)	12 (19.7)	0.805	–	–
BMI (kg/m^2^) (mean ± SD)	21.3 ± 3.1	22.7 ± 3.4	**0.030**	1.115 (0.969–1.283)	0.128
Cranial signs, n (%)	43 (87.8)	32 (52.5)	**<0.001**	**0.303 (0.101–0.909)**	**0.033**
PMR symptom	16 (32.7)	12 (19.7)	0.265	–	–
Constitutional symptoms	35 (71.4)	58 (95.1)	**0.001**	3.073 (0.685–13.791)	0.143
Limb claudication	2 (4.1)	11 (18.0)	**0.035**	3.124 (0.595–16.396)	0.178
Laboratory tests, mean ± SD					
CRP (mg/L)	102 ± 68	119 ± 56	0.175	–	–
ESR (mmHg/1st h)	89 ± 33	98 ± 29	0.137	–	–
RDW (%)	13.4 ± 1.2	13.2 ± 1.2	0.406	–	–
Albumin (g/L)	33.1 ± 3.9	30.3 ± 4.1	**<0.001**	0.865 (0.767–0.974)	0.017
Creatinine (μmol/L)	64 ± 16	66 ± 15	**0.435**	**–**	**–**

LVI, large vessel involvement; other abbreviations are the same as in Table 1. Univariate comparisons by *t*-test, Mann-Whitney U test, chi-square test, or Fisher‘s exact test. Multivariate binary logistic regression included variables with *P* < 0.10 in univariate analysis (BMI, cranial signs, constitutional symptoms, limb claudication, albumin);

*indicates variable not entered into multivariate model. Values in bold indicate statistical significance (*P* < 0.05).

### 3.3 Comparison of clinical and serologic characteristics between sex

Sex-based differences in clinical and serologic characteristics are summarized in Table 1. Compared to female patients, male patients had a shorter interval from symptom onset to first consultation (*p* = 0.013). Constitutional symptoms and morning stiffness were more prevalent in male patients. Laboratory analysis revealed that male patients had significantly lower serum albumin and higher levels of creatinine and alkaline phosphatase (ALP). The red cell distribution width (RDW) was also significantly higher in male patients (*p* = 0.043). No significant differences were found in other recorded clinical manifestations between the two sexes.

### 3.4 The performance of 1990 ACR and 2022 ACR/EUALR classification criteria on the cohort

The application of classification criteria within the cohort produced diverse results, as presented in Table 4. Specifically, 69.1% (76/110) of patients met the 1990 ACR criteria, whereas 77.3% (85/110) fulfilled the 2022 ACR/EULAR criteria. Among these patients, 70 individuals satisfied both sets of criteria. The remaining 25 patients fulfilled neither of the criteria and were diagnosed through comprehensive evaluation by experienced clinicians and a follow-up period of at least 6 months. Compared to the older criteria, the 2022 ACR/EULAR criteria captured more cases with LVI, with rates of LV-GCA increasing from 10.5% to 12.9% and mixGCA from 30.3% to 36.5%.

**TABLE 4:** The performance of 1990 ACR and 2022 ACR/EULAR classification criteria.

1990 ACR criteria	*N* (%)	2022 ACR/EULAR criteria		*N* (%)
Age ≥ 50 years	110	Absolute requirement	Age ≥ 50 years at the time of diagnosis	110
		Additional clinical criteria	Morning stiffness in shoulder/neck	32
		Sudden visual loss	2	
		Jaw and tongue claudication	21	
New onset of or new type of localized pain in the head	77		New temporal headache	77
			Scalp tenderness	31
			Abnormal examination of the temporal artery	31
Abnormal temporal artery palpation tenderness, decreased pulse	31	Laboratory, imaging, and biopsy criteria	Maximum ESR ≥ 50 mm/h or maximum CRP ≥ 10 mg/liter	100
			Positive temporal artery biopsy or halo sign on temporal artery ultrasound	46
ESR > 50 mm/h	95		Bilateral axial involvement	24
Abnormal artery biopsy	14		FDG-PET activity throughout the aorta	33
Number of patients fulfilling 1990 ACR criteria (%)	76 (69.1)	Number of patients fulfilling 2022 ACR/EULAR criteria		85 (77.3)
cGCA	45 (59.2)	cGCA		43 (50.6)
LV-GCA	8 (10.5)	LV-GCA		11 (12.9)
mixGCA	23 (30.3)	mixGCA		31 (36.5)

### 3.5 The PLR, NLR, and SII are related to ESR/CRP

Correlations between novel hematological indices and conventional inflammatory markers were assessed in Table 5. The NLR and PLR both showed significant positive correlations with CRP levels (*p* = 0.003 and *p* = 0.024, respectively). The PLR was also positively correlated with the ESR (*p* = 0.024). The SII correlated positively with CRP (*r* = 0.246, *p* = 0.010) but not with ESR (*r* = 0.184, *p* = 0.056). In contrast, serum albumin levels were inversely correlated with both ESR (*r* = −0.424, *p* < 0.001) and CRP (*r* = −0.341, *p* < 0.001).

**TABLE 5:** Correlations between inflammatory biomarkers and acute-phase reactants.

	ESR		CRP	
Biomarker	r_*s*_	*P-*value	r_*s*_	*P*-value
NLR	0.075	0.442	0.286	**0.003**
PLR	0.369	**0.024**	0.217	**0.024**
SII	0.184	0.056	0.246	**0.010**
RDW (%)	0.090	0.359	0.251	**0.009**
Albumin	−0.424	**<0.001**	**−0.341**	**<0.001**
Creatinine	0.062	0.521	0.248	**0.009**

NLR, neutrophil-to-lymphocyte ratio; PLR, platelet-to-lymphocyte ratio; SII, systemic immune-inflammation index. Values are Spearman’s correlation coefficients (r_*s*_). Values in bold indicate statistical significance (*P* < 0.05).

### 3.6 Differences in giant cell arteritis patients by lateral femoral circumflex artery (LFCA) ultrasound findings

Patients with a positive LFCA ultrasound had a significantly higher prevalence of LVI compared to those with a negative scan (100% vs 38.8%; *p* < 0.001). Additionally, the proportion of current smokers was higher in the LFCA-positive group (30.4% vs. 8.2%, *p* = 0.049), consistent with patterns observed in axillary artery ultrasound studies (Table 6).

**TABLE 6:** Differences in giant cell arteritis patients by lateral femoral circumflex artery ultrasound findings.

LFCA ultrasound findings	Negative (*n* = 49)	Positive (*n* = 23)	*P-*value
Age, mean ± SD	72 ± 7	69 ± 8	0.088
Male, n (%)	25	16	0.138
Typing (cranial/LV/mixed)	30/8/11	0/5/18	**<0.001**
Time from symptom onset to diagnosis (months)	2.74 ± 3.81	1.94 ± 1.44	0.090
Current smoker, *n* (%)	4 (8.2)	7 (30.4)	**0.014**
BMI (kg/m^2^) (mean ± SD)	21.99 ± 3.29	22.44 ± 2.71	0.572
Cranial symptoms, *n* (%)	34 (69.4)	14 (60.9)	0.475
Morning stiffness, *n* (%)	5 (10.2)	3 (13.0)	0.721
PMR symptom, *n* (%)	11 (22.4)	7 (30.4)	0.466
Constitutional symptoms, *n* (%)	43 (87.8)	22 (95.7)	0.418
Limb claudication, *n* (%)	3 (6.1)	8 (34.8)	**0.002**
Positive US of cranial arteries, *n* (%)	19/48 (39.6)	9/23 (39.1)	1.000
Positive US of axillary arteriesa, *n* (%)	4/49 (8.2)	7/23 (30.4)	**0.049**
Positive PET/CT findings, *n* (%)	24/28 (85.7)	12/12 (100)	0.548
Laboratory tests, mean ± SD			
CRP (mg/L)	111 ± 66	115 ± 60	0.825
ESR (mmHg/1st h)	93 ± 33	94 ± 21	0.855
Hemoglobin, g/L	107 ± 17	108 ± 14	0.949
RDW (%)	13.5 ± 1.4	12.9 ± 1.1	0.059
Platelet count ( × 10^9^/L)	352 ± 122	400 ± 145	0.152
NLR	7.06 ± 7.29	6.33 ± 3.93	0.653
PLR	311.61 ± 194.96	315.41 ± 128.90	0.933
SII	2704.89 ± 3327.35	2379.55 ± 1383.48	0.655
Albumin (g/L)	32.6 ± 4.1	29.9 ± 4.2	**0.011**
Globulin (g/L)	33.7 ± 5.7	34.1 ± 6.7	0.771
Creatinine (μmol/L)	64 ± 13	69 ± 20	0.246
Uric acid (μmol/L)	240 ± 78	228 ± 102	0.632
ALP (U/L)	137 ± 76	167 ± 153	0.275
Total cholesterol (mmol/L)	4.10 ± 1.17	3.60 ± 0.64	0.092

LFCA, lateral femoral circumflex artery; positive LFCA ultrasound findings: defined as the presence of a hypoechoic halo sign and/or compression sign in ultrasound scan; other abbreviations are the same as in Table 1. Statistical tests: continuous variables were analyzed using Kruskal-Wallis H test as appropriate; categorical variables were analyzed using Fisher‘s exact test. Values in bold indicate statistical significance (*P* < 0.05).

## 4 Discussion

This comprehensive investigation characterizes the clinical phenotypic spectrum of GCA within an Asian cohort, with a specific emphasis on LVI. Our findings disclosed notable demographic and clinical disparities between GCA subtypes, highlighting the necessity for refined diagnostic methodologies to facilitate the early identification of LVI.

In this cohort, the distribution of GCA subtypes was in accordance with that reported from European populations, with cGCA remaining the predominant phenotype (10, 11). Nevertheless, the overall incidence of LVI (including both isolated LV-GCA and mixGCA) was higher than that reported in European and North American cohorts (5). Such regional phenotypic divergence may be informative for local diagnostic and therapeutic strategies.

According to the literature, the onset of LVI was nearly 5 years earlier in other cohorts (12, 13). Yet in our cohort, it lacked statistical significance (*p* = 0.071) among the groups, though a trend of difference in onset age was observed. Furthermore, although diagnoses of LV-GCA and mixGCA phenotypes were frequently delayed relative to the cranial phenotype (11, 12), this difference was not displayed in our cohort. Such discrepancy may be attributable to differences in study populations.

Clinically, constitutional symptoms and limb claudication were significantly more prevalent in the LVI group, reinforcing limb claudication as a suggestive feature of LVI (13). In contrast to European cohorts (10–12), LVI was not associated with female sex or concomitant PMR in our Asian cohort.

In clinical practice, recognition of LVI is of great importance. From a long-term perspective, LVI was the strongest predictor of aortic dilation (hazard ratio: 3.16), necessitating vigilant monitoring (10). In our cohort, univariate analysis revealed significant differences between patients with and without LVI across several factors, consistent with previous reports (13). Notably, serum albumin was independently associated with LVI in the multivariate model (OR = 0.865, *p* = 0.017). We hypothesized that hypoalbuminemia may serve as a surrogate marker for more severe systemic inflammation in the LVI subgroup of patients. Nevertheless, the discrepancy may stem from differences in population characteristics, sample size, or the definition of LVI subtypes. Further validation in larger cohort studies is warranted to confirm the reliability of our conclusion.

Diagnostically, the halo sign on temporal artery ultrasound (TA-US) confers considerable diagnostic value in GCA. Despite our study having enrolled patients as early as 2015, the halo sign showed a high rate of concordance with the newly proposed definitions by an Outcome Measures in Rheumatology (OMERACT) working group (14).

In our cohort, the positivity rate of TA-US was lower than that observed in a Norwegian cohort (12). Patient selection bias in our cohort may partially explain this difference. Firstly, TA-US examination was not performed in some patients who had positive PET-CT findings for cranial arteries. Secondly, we systematically screened for patients with isolated LV-GCA, a subgroup that typically presents with negative US. This may further contribute to the lower US positivity rate. Our study also indicated that bilateral TA-US evaluation improved diagnostic sensitivity as the meta-analysis reported (15).

In the routine US scan of LV-GCA, the positivity rate of the axillary artery was aligned with previously reported rates, which range from 5% to 31% (16). In our cohort, a positive LFCA ultrasound finding was strongly associated with large-vessel involvement, limb claudication, and current smoking, suggesting that smoking may be a confounder or risk factor for vascular changes in this population. Given the limited sensitivity of routine scanning for LV-GCA, adding LFCA evaluation may increase diagnostic yield. Nonetheless, positive findings should be interpreted with caution, for the atherosclerosis can mimic vasculitis (17).

Regarding advanced imaging, the sensitivity of [18F]FDG PET-CT varied depending on the arteries involved. Its pooled sensitivity drops from 60–77% for cranial vasculitis to 20–60% for large-vessel vasculitis (13, 18). Emerging hybrid imaging such as PET-MRI fusion allows simultaneous assessment of mural inflammation and luminal changes, though standardized quantification protocols are currently lacking (19). Therefore, an integrated protocol combining extended ultrasound with PET-CT or PET-MRI should be considered in future studies. Finally, ultrasound guidance did not increase the TAB positivity rate beyond the reported 50% (20).

We validated the 1990 ACR classification criteria and the 2022 ACR/EULAR criteria in our cohort, with fulfillment rates of 69.1% and 77.3%, respectively. However, their performance varied across subtypes. Both criteria showed consistent sensitivity for cGCA (59.2% and 50.6%, respectively). Our cohort had higher detection rates for LV-GCA and mixGCA using the 2022 ACR/EULAR criteria, while lower sensitivity was observed for extracranial GCA in another cohort (5). A review concluded that the new criteria could improve sensitivity across all subtypes (13). Notably, the remaining 25 GCA patients met neither of the criteria. These patients received a clinical diagnosis based on all available information and long-term follow-up. Thus, both criteria have inherent limitations when used for clinical diagnosis.

Cerebrovascular ischemic events (CIE) are severe complications of GCA, typically occurring early in the disease courses (21, 22). While French cohort studies and a meta-analysis reported CIE prevalence ranging from 1.5% to 12.95% (23), the incidence in our cohort was only 0.91%. Several studies indicated that fever, elevated ESR/CRP levels, lower vertebral and axillary arteries involvement were negatively associated with CIE occurrence (20, 24). Given the low incidence of CIE in our cohort, no further analysis was conducted.

Regarding therapeutic strategies, the majority of patients at our center received combination therapy with glucocorticoids and DMARDs. While earlier evidence suggested that adjunctive methotrexate (MTX) could reduce relapse risk (25, 26), further validation is needed in our long-term follow-up.

When interpreting the results of our study, one must consider the potential limitations inherent in its retrospective nature and limited sample size. Furthermore, all patients were clinically diagnosed at a single tertiary center. Specifically, among the 25 unclassified patients, the diagnosis of giant cell arteritis was based on clinical judgment alone without confirmatory imaging or biopsy. Therefore, the results may not be generalizable to all patients with GCA in clinical practice. Future prospective, multicenter designs are needed to validate these findings and to establish phenotype-specific diagnostic and management approaches customized for Asian populations.

## 5 Conclusion

This research confirms the substantial phenotypic heterogeneity of GCA within an Asian cohort. Compared to cGCA, patients with LV-GCA and mixGCA had more constitutional symptoms and limb claudication but fewer cranial manifestations. The 2022 ACR/EULAR criteria showed enhanced overall sensitivity, particularly for detecting LVI. Practical adjunctive tools, including lower-extremity arterial ultrasound (e.g., LFCA) and serum albumin levels, can aid in the identification of LVI.

## Data Availability

The original contributions presented in this study are included in the article/Supplementary material, further inquiries can be directed to the corresponding author.
