# Supplementary figures and images for: Phenotypic heterogeneity of giant cell arteritis in an Asian cohort: clinical, imaging, and laboratory characteristics
Source: Front Med (Lausanne). 2026 Jun 30;13:1812045. doi: 10.3389/fmed.2026.1812045 (PMC13364556; doi:10.3389/fmed.2026.1812045)

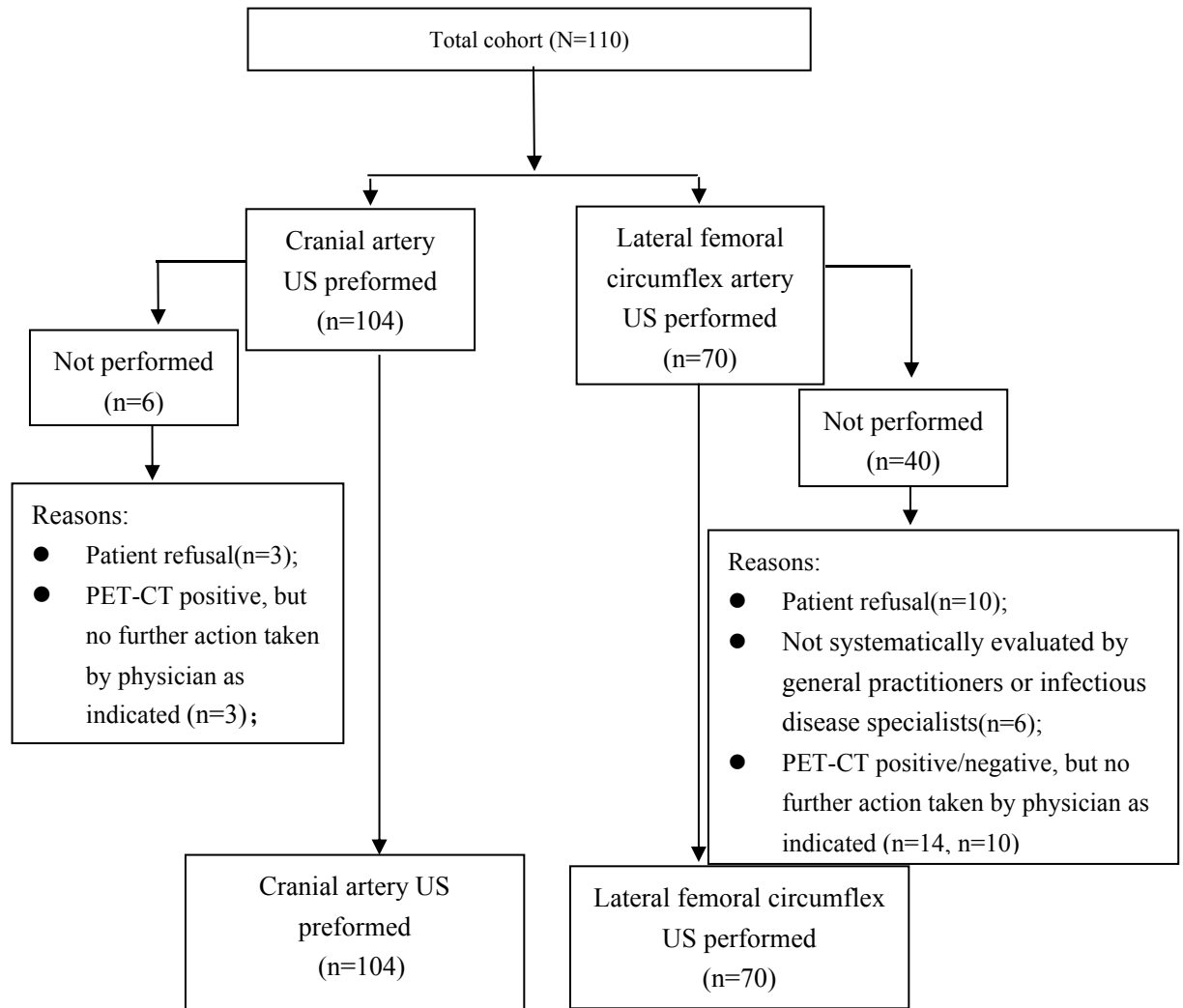

Supplement: Supplementary file 1 [file Image_1.pdf]
